# (4-Hydr­oxy-1,1-dioxo-2*H*-1,2-benzothia­zin-3-yl)(3-methoxy­phen­yl)methanone

**DOI:** 10.1107/S1600536810011827

**Published:** 2010-04-02

**Authors:** Salman Gul, Hamid Latif Siddiqui, Matloob Ahmad, Masood Parvez

**Affiliations:** a Institute of Chemistry, University of the Punjab, Lahore 54590, Pakistan; b Applied Chemistry Research Centre, PCSIR Laboratories Complex, Lahore 54600, Pakistan; c Department of Chemistry, The University of Calgary, 2500 University Drive NW, Calgary, Alberta, Canada T2N 1N4

## Abstract

In the title compoud, C_16_H_13_NO_5_S, the heterocyclic thia­zine ring adopts a twist boat conformation with the S and N atoms displaced by 0.339 (5) and 0.322 (4) Å, respectively, on opposite sides of the mean plane formed by the remaining ring atoms. An intra­molecular O—H⋯O inter­action is present, forming a five-membered ring. The crystal structure is stabilized by inter­molecular N—H⋯O hydrogen bonds, which result in chains along the *b* axis.

## Related literature

For the biological activity of 1,2-benzothia­zine derivatives, see: Ikeda *et al.* (1992 ▶); Ahmad *et al.* (2010 ▶); Lombardino *et al.* (1971 ▶, 1973 ▶); Zia-ur-Rehman *et al.* (2006 ▶); Siddiqui *et al.* (2007 ▶). For comparison bond lengths, see: Allen *et al.* (1987 ▶). For related structures, see: Siddiqui *et al.* (2008 ▶).
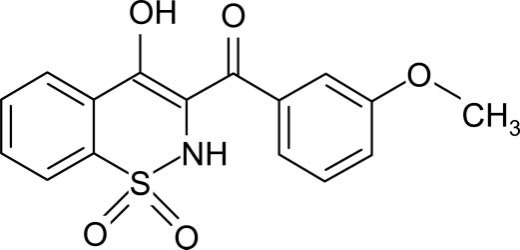


## Experimental

### 

#### Crystal data

C_16_H_13_NO_5_S
*M* *_r_* = 331.33
Monoclinic, 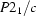

*a* = 8.1866 (3) Å
*b* = 7.2431 (3) Å
*c* = 25.2452 (9) Å
β = 95.5869 (18)°
*V* = 1489.84 (10) Å^3^
*Z* = 4
Mo *K*α radiation
μ = 0.24 mm^−1^
*T* = 295 K
0.16 × 0.12 × 0.10 mm

#### Data collection

Nonius KappaCCD diffractometer
Absorption correction: multi-scan (*SORTAV*; Blessing, 1997 ▶) *T*_min_ = 0.962, *T*_max_ = 0.976
5610 measured reflections
3399 independent reflections
2795 reflections with *I* > 2σ(*I*)
*R*_int_ = 0.031

#### Refinement

*R*[*F* ^2^ > 2σ(*F* ^2^)] = 0.056
*wR*(*F* ^2^) = 0.147
*S* = 1.09
3399 reflections
215 parameters
H atoms treated by a mixture of independent and constrained refinement
Δρ_max_ = 0.38 e Å^−3^
Δρ_min_ = −0.38 e Å^−3^

### 

Data collection: *COLLECT* (Hooft, 1998 ▶); cell refinement: *DENZO* (Otwinowski & Minor, 1997 ▶); data reduction: *SCALEPACK* (Otwinowski & Minor, 1997 ▶); program(s) used to solve structure: *SHELXS97* (Sheldrick, 2008 ▶); program(s) used to refine structure: *SHELXL97* (Sheldrick, 2008 ▶); molecular graphics: *ORTEP-3 for Windows* (Farrugia, 1997 ▶); software used to prepare material for publication: *SHELXL97*.

## Supplementary Material

Crystal structure: contains datablocks global, I. DOI: 10.1107/S1600536810011827/fl2296sup1.cif

Structure factors: contains datablocks I. DOI: 10.1107/S1600536810011827/fl2296Isup2.hkl

Additional supplementary materials:  crystallographic information; 3D view; checkCIF report

## Figures and Tables

**Table 1:** Hydrogen-bond geometry (Å, °)

*D*—H⋯*A*	*D*—H	H⋯*A*	*D*⋯*A*	*D*—H⋯*A*
O3—H3*O*⋯O4	0.92 (3)	1.70 (3)	2.534 (2)	148 (3)
N1—H1*N*⋯O4^i^	0.84 (3)	2.13 (3)	2.886 (3)	151 (3)

Symmetry code: (i) 
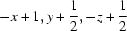
.

**Fig. 1.**
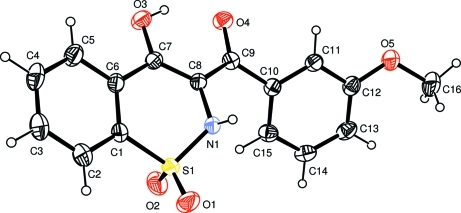
The title molecule with the displacement ellipsoids plotted at 30% probability level (Farrugia, 1997).

**Fig. 2.**
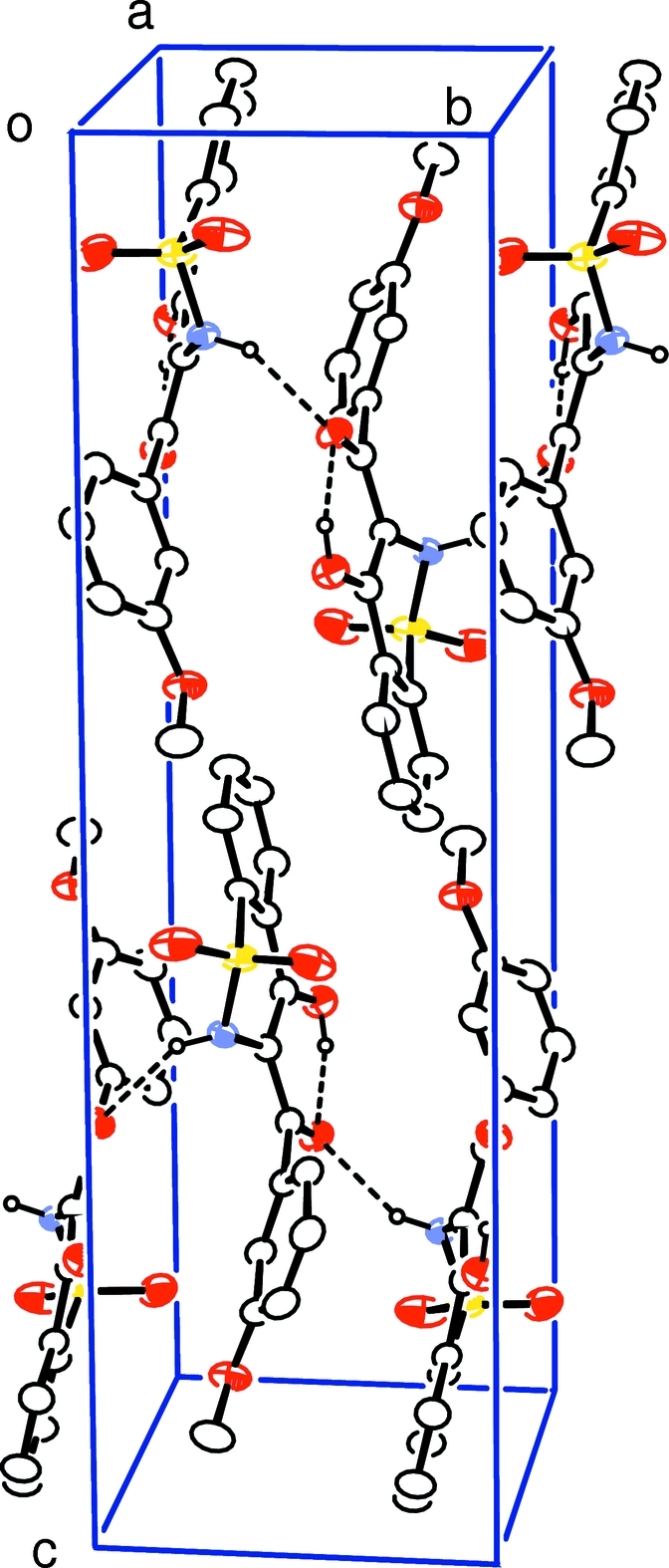
A unit cell showing molecular packing of the title compound; hydrogen bonds are represented by dashed lines. The H-atoms not involved in H-bonds have been excluded for clarity.

## supplementary crystallographic information

### Comment

Benzothiazine dioxide derivatives have been extensively explored in the past few decades since their very first derivatives were found to be potent anti-inflammatory and analgesic agents (Lombardino *et al.*, 1971). Benzothiazines derivatives are now known to be anti-allergy (Ikeda *et al.*, 1992), anti-inflammatory (Lombardino *et al.*, 1973), bactericidal (Zia-ur-Rehman *et al.*, 2006), etc. In continuation of our research on benzothiazine compounds (Ahmad *et al.*, 2010, Siddiqui *et al.*, 2007), we report the synthesis and crystal structure of the title compound (I) in this paper  (Fig. 1).

Bond distances (Allen *et al.*, 1987) and angles are as expected and agree with the corresponding bond distances and angles reported in closely related compounds (Siddiqui *et al.*, 2008). The heterocyclic thiazine ring adopts a twist boat conformation with atoms S1 and N1 displaced by 0.339 (5) and 0.322 (4) Å , respectively, on the opposite sides from the mean plane formed by the remaining ring atoms.

The structure is stabilized by N—H···O type intermolecular hydrogen bonds which result in one dimensional chains of molecules extended along the *b*-axis; intramolecular interactions O3—H3O···O4 are also present resulting in five membered rings (Table 1 and Fig. 2).

### Experimental

2-[2-(3-Methoxyphenyl)-2-oxoethyl]-1,2-benzisothiazol-3(2*H*)-one 1,1-dioxide (5.0 g, 15.1 mmoles) was added to a solution of sodium metal (2.4 g) in dry methanol (50 ml). The mixture was subjected to reflux for half an hour. The contents of the flask were cooled to room temperature and then they were poured on ice cold HCl (50 ml, 5%). Light yellow precipitates of the title compound  formed which were filtered off and  washed with excess distilled water. Crystals suitable for XRD were grown in chloroform and methanol mixture (4:1). Yield = 3.7 g, 74%; m.p. = 425-427 K.

### Refinement

Though all the H atoms could be distinguished in the difference Fourier map the H-atoms bonded to C-atoms were included at geometrically idealized positions and refined in riding-model approximation with the following constraints: C—H distances were set to 0.93 and 0.96 Å, for aryl and methyl H-atoms, respectively; the H-atoms bonded to N and O were allowed to refine. The *U*_iso_(H) were allowed at 1.2*U*_eq_(parent atom). The final difference map was essentially featurless.

### Crystal data

**Table:**

C_16_H_13_NO_5_S	*F*(000) = 688
*M**_r_* = 331.33	*D*_x_ = 1.477 Mg m^−^^3^
Monoclinic, *P*2_1_/*c*	Mo *K*α radiation, λ = 0.71073 Å
Hall symbol: -P 2ybc	Cell parameters from 3461 reflections
*a* = 8.1866 (3) Å	θ = 1.0–27.5°
*b* = 7.2431 (3) Å	µ = 0.24 mm^−^^1^
*c* = 25.2452 (9) Å	*T* = 295 K
β = 95.5869 (18)°	Block, yellow
*V* = 1489.84 (10) Å^3^	0.16 × 0.12 × 0.10 mm
*Z* = 4	

### Data collection

**Table:**

Nonius KappaCCD diffractometer	3399 independent reflections
Radiation source: fine-focus sealed tube	2795 reflections with *I* > 2σ(*I*)
graphite	*R*_int_ = 0.031
ω and φ scans	θ_max_ = 27.5°, θ_min_ = 2.9°
Absorption correction: multi-scan (*SORTAV*; Blessing, 1997)	*h* = −10→10
*T*_min_ = 0.962, *T*_max_ = 0.976	*k* = −9→9
5610 measured reflections	*l* = −32→32

### Refinement

**Table:**

Refinement on *F*^2^	Primary atom site location: structure-invariant direct methods
Least-squares matrix: full	Secondary atom site location: difference Fourier map
*R*[*F*^2^ > 2σ(*F*^2^)] = 0.056	Hydrogen site location: difference Fourier map
*wR*(*F*^2^) = 0.147	H atoms treated by a mixture of independent and constrained refinement
*S* = 1.09	*w* = 1/[σ^2^(*F*_o_^2^) + (0.051*P*)^2^ + 1.1474*P*] where *P* = (*F*_o_^2^ + 2*F*_c_^2^)/3
3399 reflections	(Δ/σ)_max_ < 0.001
215 parameters	Δρ_max_ = 0.38 e Å^−^^3^
0 restraints	Δρ_min_ = −0.38 e Å^−^^3^

### Special details

**Table:**

Geometry. All esds (except the esd in the dihedral angle between two l.s. planes) are estimated using the full covariance matrix. The cell esds are taken into account individually in the estimation of esds in distances, angles and torsion angles; correlations between esds in cell parameters are only used when they are defined by crystal symmetry. An approximate (isotropic) treatment of cell esds is used for estimating esds involving l.s. planes.
Refinement. Refinement of *F*^2^ against ALL reflections. The weighted *R*-factor wR and goodness of fit *S* are based on *F*^2^, conventional *R*-factors *R* are based on *F*, with *F* set to zero for negative *F*^2^. The threshold expression of *F*^2^ > σ(*F*^2^) is used only for calculating *R*-factors(gt) etc. and is not relevant to the choice of reflections for refinement. *R*-factors based on *F*^2^ are statistically about twice as large as those based on *F*, and *R*- factors based on ALL data will be even larger.

### Fractional atomic coordinates and isotropic or equivalent isotropic displacement parameters (Å^2^)

**Table:**

	*x*	*y*	*z*	*U*_iso_*/*U*_eq_	
S1	0.30794 (7)	0.18400 (11)	0.10208 (2)	0.0519 (2)	
O1	0.1907 (2)	0.3174 (4)	0.08123 (8)	0.0825 (8)	
O2	0.2664 (3)	−0.0070 (3)	0.09981 (8)	0.0780 (7)	
O3	0.7829 (2)	0.0710 (3)	0.18344 (8)	0.0532 (5)	
H3O	0.772 (4)	0.058 (4)	0.2194 (12)	0.064*	
O4	0.6461 (2)	0.0579 (3)	0.26917 (7)	0.0502 (4)	
O5	0.2363 (2)	0.2043 (3)	0.40084 (7)	0.0639 (6)	
N1	0.3602 (2)	0.2363 (3)	0.16357 (7)	0.0405 (4)	
H1N	0.350 (3)	0.347 (4)	0.1721 (11)	0.049*	
C1	0.4921 (3)	0.2116 (3)	0.07263 (9)	0.0443 (5)	
C2	0.4907 (4)	0.2573 (4)	0.01952 (10)	0.0608 (7)	
H2	0.3919	0.2803	−0.0008	0.073*	
C3	0.6372 (4)	0.2684 (5)	−0.00311 (11)	0.0669 (8)	
H3	0.6374	0.2979	−0.0390	0.080*	
C4	0.7820 (4)	0.2361 (5)	0.02714 (12)	0.0686 (8)	
H4	0.8802	0.2442	0.0116	0.082*	
C5	0.7853 (3)	0.1919 (4)	0.08025 (11)	0.0561 (7)	
H5	0.8850	0.1703	0.1002	0.067*	
C6	0.6392 (3)	0.1796 (3)	0.10413 (9)	0.0415 (5)	
C7	0.6420 (3)	0.1356 (3)	0.16109 (9)	0.0396 (5)	
C8	0.5064 (3)	0.1554 (3)	0.18882 (8)	0.0382 (5)	
C9	0.5114 (3)	0.0999 (3)	0.24377 (9)	0.0407 (5)	
C10	0.3583 (3)	0.0867 (3)	0.27107 (9)	0.0399 (5)	
C11	0.3629 (3)	0.1488 (3)	0.32355 (9)	0.0433 (5)	
H11	0.4583	0.2005	0.3403	0.052*	
C12	0.2229 (3)	0.1324 (4)	0.35052 (9)	0.0485 (6)	
C13	0.0846 (3)	0.0446 (4)	0.32655 (11)	0.0550 (7)	
H13	−0.0078	0.0308	0.3449	0.066*	
C14	0.0844 (3)	−0.0224 (4)	0.27528 (11)	0.0529 (6)	
H14	−0.0076	−0.0845	0.2598	0.063*	
C15	0.2183 (3)	0.0011 (3)	0.24657 (10)	0.0469 (5)	
H15	0.2150	−0.0395	0.2115	0.056*	
C16	0.0876 (4)	0.2197 (6)	0.42631 (13)	0.0785 (10)	
H16A	0.1089	0.2871	0.4590	0.094*	
H16B	0.0480	0.0985	0.4337	0.094*	
H16C	0.0063	0.2837	0.4032	0.094*	

### Atomic displacement parameters (Å^2^)

**Table:**

	*U*^11^	*U*^22^	*U*^33^	*U*^12^	*U*^13^	*U*^23^
S1	0.0374 (3)	0.0828 (5)	0.0356 (3)	−0.0035 (3)	0.0039 (2)	−0.0057 (3)
O1	0.0477 (11)	0.150 (2)	0.0493 (11)	0.0280 (13)	0.0017 (8)	0.0181 (13)
O2	0.0722 (14)	0.0964 (17)	0.0683 (13)	−0.0391 (13)	0.0216 (11)	−0.0321 (12)
O3	0.0387 (9)	0.0667 (12)	0.0545 (10)	0.0061 (8)	0.0054 (8)	0.0077 (9)
O4	0.0439 (9)	0.0615 (11)	0.0446 (9)	0.0019 (8)	0.0011 (7)	0.0117 (8)
O5	0.0547 (11)	0.0942 (16)	0.0447 (10)	0.0029 (10)	0.0150 (8)	−0.0049 (10)
N1	0.0389 (10)	0.0488 (11)	0.0340 (9)	0.0058 (9)	0.0049 (7)	−0.0035 (8)
C1	0.0439 (12)	0.0530 (14)	0.0371 (11)	−0.0021 (10)	0.0091 (9)	−0.0066 (10)
C2	0.0648 (17)	0.080 (2)	0.0387 (12)	0.0017 (15)	0.0090 (12)	−0.0008 (13)
C3	0.082 (2)	0.079 (2)	0.0431 (13)	0.0001 (17)	0.0238 (14)	0.0016 (14)
C4	0.0667 (18)	0.085 (2)	0.0598 (17)	−0.0013 (16)	0.0357 (15)	0.0012 (16)
C5	0.0465 (14)	0.0649 (17)	0.0596 (15)	−0.0009 (12)	0.0182 (12)	−0.0007 (13)
C6	0.0422 (12)	0.0427 (12)	0.0411 (11)	−0.0013 (10)	0.0112 (9)	−0.0045 (9)
C7	0.0359 (11)	0.0392 (11)	0.0438 (11)	0.0000 (9)	0.0051 (9)	−0.0015 (9)
C8	0.0367 (11)	0.0422 (11)	0.0357 (10)	0.0031 (9)	0.0032 (8)	−0.0006 (9)
C9	0.0429 (12)	0.0388 (11)	0.0404 (11)	0.0004 (9)	0.0040 (9)	0.0012 (9)
C10	0.0414 (12)	0.0406 (11)	0.0380 (11)	0.0027 (9)	0.0053 (9)	0.0060 (9)
C11	0.0399 (12)	0.0490 (13)	0.0411 (11)	0.0017 (10)	0.0043 (9)	0.0062 (10)
C12	0.0480 (13)	0.0568 (15)	0.0417 (12)	0.0070 (11)	0.0096 (10)	0.0082 (11)
C13	0.0397 (13)	0.0709 (18)	0.0553 (15)	0.0020 (12)	0.0093 (11)	0.0165 (13)
C14	0.0392 (12)	0.0593 (16)	0.0585 (15)	−0.0052 (11)	−0.0035 (11)	0.0105 (12)
C15	0.0489 (13)	0.0491 (13)	0.0418 (12)	−0.0015 (11)	−0.0002 (10)	0.0061 (10)
C16	0.069 (2)	0.106 (3)	0.0654 (19)	0.0085 (19)	0.0307 (16)	−0.0085 (18)

### Geometric parameters (Å, °)

**Table:**

S1—O2	1.425 (2)	C5—C6	1.394 (3)
S1—O1	1.426 (2)	C5—H5	0.9300
S1—N1	1.6145 (19)	C6—C7	1.471 (3)
S1—C1	1.756 (2)	C7—C8	1.377 (3)
O3—C7	1.319 (3)	C8—C9	1.441 (3)
O3—H3O	0.92 (3)	C9—C10	1.491 (3)
O4—C9	1.257 (3)	C10—C15	1.394 (3)
O5—C12	1.367 (3)	C10—C11	1.396 (3)
O5—C16	1.436 (3)	C11—C12	1.394 (3)
N1—C8	1.426 (3)	C11—H11	0.9300
N1—H1N	0.84 (3)	C12—C13	1.385 (4)
C1—C2	1.380 (3)	C13—C14	1.382 (4)
C1—C6	1.396 (3)	C13—H13	0.9300
C2—C3	1.381 (4)	C14—C15	1.382 (3)
C2—H2	0.9300	C14—H14	0.9300
C3—C4	1.366 (4)	C15—H15	0.9300
C3—H3	0.9300	C16—H16A	0.9600
C4—C5	1.376 (4)	C16—H16B	0.9600
C4—H4	0.9300	C16—H16C	0.9600
			
O2—S1—O1	119.57 (16)	C8—C7—C6	122.5 (2)
O2—S1—N1	107.85 (12)	C7—C8—N1	119.92 (19)
O1—S1—N1	107.56 (13)	C7—C8—C9	120.8 (2)
O2—S1—C1	107.77 (12)	N1—C8—C9	119.30 (19)
O1—S1—C1	109.96 (13)	O4—C9—C8	120.0 (2)
N1—S1—C1	102.85 (11)	O4—C9—C10	118.9 (2)
C7—O3—H3O	107.2 (19)	C8—C9—C10	121.1 (2)
C12—O5—C16	116.8 (2)	C15—C10—C11	120.6 (2)
C8—N1—S1	117.75 (15)	C15—C10—C9	121.0 (2)
C8—N1—H1N	112.5 (19)	C11—C10—C9	118.1 (2)
S1—N1—H1N	116.6 (19)	C12—C11—C10	119.2 (2)
C2—C1—C6	121.2 (2)	C12—C11—H11	120.4
C2—C1—S1	120.8 (2)	C10—C11—H11	120.4
C6—C1—S1	117.92 (17)	O5—C12—C13	124.7 (2)
C1—C2—C3	119.4 (3)	O5—C12—C11	115.2 (2)
C1—C2—H2	120.3	C13—C12—C11	120.1 (2)
C3—C2—H2	120.3	C14—C13—C12	119.8 (2)
C4—C3—C2	120.0 (3)	C14—C13—H13	120.1
C4—C3—H3	120.0	C12—C13—H13	120.1
C2—C3—H3	120.0	C15—C14—C13	121.3 (2)
C3—C4—C5	121.2 (3)	C15—C14—H14	119.3
C3—C4—H4	119.4	C13—C14—H14	119.3
C5—C4—H4	119.4	C14—C15—C10	118.8 (2)
C4—C5—C6	120.0 (3)	C14—C15—H15	120.6
C4—C5—H5	120.0	C10—C15—H15	120.6
C6—C5—H5	120.0	O5—C16—H16A	109.5
C5—C6—C1	118.2 (2)	O5—C16—H16B	109.5
C5—C6—C7	120.3 (2)	H16A—C16—H16B	109.5
C1—C6—C7	121.6 (2)	O5—C16—H16C	109.5
O3—C7—C8	122.3 (2)	H16A—C16—H16C	109.5
O3—C7—C6	115.16 (19)	H16B—C16—H16C	109.5
			
O2—S1—N1—C8	66.4 (2)	C6—C7—C8—N1	−5.7 (3)
O1—S1—N1—C8	−163.37 (18)	O3—C7—C8—C9	−2.0 (4)
C1—S1—N1—C8	−47.3 (2)	C6—C7—C8—C9	176.1 (2)
O2—S1—C1—C2	94.1 (3)	S1—N1—C8—C7	39.2 (3)
O1—S1—C1—C2	−37.8 (3)	S1—N1—C8—C9	−142.49 (19)
N1—S1—C1—C2	−152.2 (2)	C7—C8—C9—O4	10.3 (4)
O2—S1—C1—C6	−83.7 (2)	N1—C8—C9—O4	−167.9 (2)
O1—S1—C1—C6	144.4 (2)	C7—C8—C9—C10	−168.2 (2)
N1—S1—C1—C6	30.0 (2)	N1—C8—C9—C10	13.6 (3)
C6—C1—C2—C3	1.1 (4)	O4—C9—C10—C15	−133.0 (2)
S1—C1—C2—C3	−176.6 (2)	C8—C9—C10—C15	45.5 (3)
C1—C2—C3—C4	−0.6 (5)	O4—C9—C10—C11	41.8 (3)
C2—C3—C4—C5	0.1 (5)	C8—C9—C10—C11	−139.7 (2)
C3—C4—C5—C6	−0.1 (5)	C15—C10—C11—C12	−3.1 (4)
C4—C5—C6—C1	0.5 (4)	C9—C10—C11—C12	−177.9 (2)
C4—C5—C6—C7	−178.9 (3)	C16—O5—C12—C13	−12.1 (4)
C2—C1—C6—C5	−1.1 (4)	C16—O5—C12—C11	169.3 (3)
S1—C1—C6—C5	176.7 (2)	C10—C11—C12—O5	−177.2 (2)
C2—C1—C6—C7	178.4 (2)	C10—C11—C12—C13	4.2 (4)
S1—C1—C6—C7	−3.8 (3)	O5—C12—C13—C14	179.8 (2)
C5—C6—C7—O3	−14.2 (3)	C11—C12—C13—C14	−1.7 (4)
C1—C6—C7—O3	166.3 (2)	C12—C13—C14—C15	−1.9 (4)
C5—C6—C7—C8	167.5 (2)	C13—C14—C15—C10	3.0 (4)
C1—C6—C7—C8	−11.9 (4)	C11—C10—C15—C14	−0.4 (4)
O3—C7—C8—N1	176.2 (2)	C9—C10—C15—C14	174.2 (2)

### Hydrogen-bond geometry (Å, °)

**Table:**

*D*—H···*A*	*D*—H	H···*A*	*D*···*A*	*D*—H···*A*
O3—H3O···O4	0.92 (3)	1.70 (3)	2.534 (2)	148 (3)
N1—H1N···O4^i^	0.84 (3)	2.13 (3)	2.886 (3)	151 (3)

Symmetry codes: (i) −*x*+1, *y*+1/2, −*z*+1/2.
